# Correlation between lower balance of Th2 helper T-cells and expression of PD-L1/PD-1 axis genes enables prognostic prediction in patients with glioblastoma

**DOI:** 10.18632/oncotarget.24897

**Published:** 2018-04-10

**Authors:** Yasuo Takashima, Atsushi Kawaguchi, Tomohiko Kanayama, Azusa Hayano, Ryuya Yamanaka

**Affiliations:** ^1^ Laboratory of Molecular Target Therapy for Cancer, Graduate School of Medical Science, Kyoto Prefectural University of Medicine, Kyoto 602-8566, Japan; ^2^ Center for Comprehensive Community Medicine, Faculty of Medicine, Saga University, Saga 849-8501, Japan

**Keywords:** glioblastoma, prognosis, helper T-cells, PD-L1, PD-1

## Abstract

Common cancer treatments include radiation therapy, chemotherapy including molecular targeted drugs and anticancer drugs, and surgical treatment. Recent studies have focused on investigating the mechanisms by which immune cells attack cancer cells and produce immune tolerance-suppressing cytokines, as well as on their potential application in cancer immunotherapy. We conducted expression profiling of *CD274* (*PD-L1*), *GATA3, IFNG, IL12R, IL12RB2, IL4, PDCD1* (*PD-1*), *PDCD1LG2* (*PD-L2*), and *TBX21* (*T-bet*) using data of 158 glioblastoma multiforme (GBM) patients with clinical information available at The Cancer Genome Atlas. Principal component analysis of the expression profiling data was used to derive an equation for evaluating the status of Th1 and Th2 cells. GBM specimens were divided based on the median of the Th scores. The results revealed that Th1^High^Th2^Low^ and Th1^Low^Th2^Low^ statuses indicated better prognosis than Th1^High^Th2^High^, and were evaluated based on the downregulation of PD-L1, PD-L2, and PD-1. Furthermore, Th2^Low^ divided based on the threshold, as well as CD274^Low^ and PDCD1^Low^, were associated with good prognosis. In the Th2^Low^ subgroup, 14 genes were identified as potential prognostic markers. Of these, SLC11A1^Low^, TNFRSF1B^Low^, and LTBR^Low^ also indicated good prognosis. These results suggest that low Th2 balance and low activity of the PD-L1/PD-1 axis predict good prognosis in GBM. The set of genes identified in the present study could reliably predict survival in GBM patients and serve as useful molecular markers. Furthermore, this set of genes could prove to be novel targets for cancer immunotherapy.

## INTRODUCTION

Gliomas are the most common type of primary central nervous system (CNS) tumors and represent 40% of brain tumors [1]. The World Health Organization (WHO) has classified gliomas into grades I–IV by the order of increasing malignancy and decreasing overall survival (OS) [2,3]. Glioblastoma multiforme (GBM) is a grade IV, fast-growing type of malignant glioma. It is the most common brain tumor affecting adults, with a median survival period of only 9–15 months [4, 5]. Therefore, GBM is considered the most malignant and aggressive form of primary brain tumor. GBM has an overall 5-year survival rate of only 9.8% even after treatment via surgery, radiotherapy, or chemotherapy [4, 6]. Thus, early diagnosis and treatment of GBM is critical for predicting an accurate prognosis. In other words, more effective therapeutic strategies, a more detailed understanding of the biological mechanisms underlying GBM, and the identification of novel molecular targets are required for improved diagnosis and therapies for GBM.

Recent studies have targeted CNS tumors for cancer immunotherapy, and this approach has yielded progress in neurobiology, oncology, and immunology in malignant gliomas [7]. Cancer immunotherapy targets immune checkpoint molecules located on the surface of antigen-presenting cells (APCs), glioma cells, and helper T-cells. For example, researchers have developed monoclonal antibody therapies that target programmed cell death protein-1 (PD-1), cytotoxic T lymphocyte-associated antigen-4 (CTLA-4), and indoleamine 2,3-dioxygenase (IDO) as reliable checkpoint molecules for cancer immunotherapy [8]. PD-1 signaling occurs during the effector phase of the immune response within tumor microenvironments. The inhibitory PD-1 receptor, which is expressed on the surface of T-cells, interacts with PD-1 ligands, including PD-L1 and PD-L2, which are expressed on the surfaces of tumor cells. In the context of tumor major histocompatibility complex (MHC) class I antigen presentation, ligand interactions with PD-1 are known to inhibit T-cell tumor lytic capacity and induce T-cell anergy [9]. CTLA 4 is a highly potent inhibitory T-cell receptor; it preferentially binds to B7-1 (cluster of differentiation (CD)80) and B7-2 (CD86) receptors on the surface of APCs. In turn, this prevents binding to the T-cell receptor, which triggers the production of interleukin-6 (IL-6), and prevents the binding of APCs to the CD28 co-stimulatory receptor on the surface of T-cells [10,11]. Such ligand–receptor interactions lead to decreased activation and proliferation of T-cells, which prevents MHC class I antigen presentation [12]. In addition, IDO-expressing cells, including dendritic cells and macrophages, have been demonstrated to regulate T-cell metabolism and response by catalyzing the oxidative catabolism of tryptophan in kynurenine (KYN) signaling [8], aryl hydrocarbon receptor (AhR), general control nonderepressible 2 (GCN2) as a Ser/Thr protein kinase, and mammalian target of rapamycin (mTOR) signaling [13].

A recent study performed expression profiling and clinical characterization of the PD-L1 gene using 301 microarray data from the Chinese Glioma Genome Atlas (CGGA) and RNA-Seq data from 675 samples of grade II–IV gliomas, including primary and secondary tumors, from The Cancer Genome Atlas (TCGA) [14]. However, it is difficult to estimate the prognoses of glioma patients in mixed stages, including grades II–IV and primary and secondary gliomas. This requires not only PD-L1 profiling but also additional analyses, such as the evaluation of Th1–Th2 balance. Naïve T-cells differentiate into Th1 and Th2 cells [15,16]. Th1 cells, which are activated by IL-2/IL-12, protect against bacteria and protozoa by producing interferon (IFN) γ. Macrophages, IFN-γ CD4^+^ T-cells, CD8^+^ T-cells, and IgG B-cells are the primary effectors of Th1 immunity. Th1 cells are identified based on TBX21 (T-bet) and STAT4 expression [15, 16]. On the other hand, Th2 cells, which are activated by IL-4, function by eliminating extracellular parasites and producing effector cytokines, including IL-4, IL-5, IL-9, IL-10, and IL-13. The effector cells of Th2 immunity mainly comprise mast cells, IL-4/IL-5 CD4^+^ T-cells, and B-cells. The key Th2 transcription factors include GATA3 and STAT6 [17]. Therefore, it is important to investigate T-cell status using appropriate gene expression profiling techniques in malignant gliomas.

In this study, we performed the gene expression profiling of CD274 (PD-L1), GATA3, IFNG, IL12R, IL12RB2, IL4, PDCD1 (PD-1), PDCD1LG2 (PD-L2), and TBX21 (T-bet) using 158 primary GBM samples from the TCGA data set, followed by principal component analysis (PCA) to obtain Th1 and Th2 scores. GBM specimens were grouped based on the calculated Th1 and Th2 scores, and patient prognosis was estimated based on the expression levels of the PD-L1/PD-1 axis genes. Furthermore, we evaluated the utility of the obtained signature genes as prognostic markers in GBM to identify patient subgroups with good prognosis and identified novel candidate prognostic markers in GBM. The genes identified in the present study may serve as novel targets for cancer immunotherapy.

## RESULTS

### Patient characteristics

The present study recruited 158 non-treated primary GBM patients (WHO grade IV) whose data were deposited in the “Glioblastoma Multiforme (TCGA, Provisional)” data set between 2008 and 2012 (Table 1, Supplementary Figure 1A). The median age of the patients was 60 years (range, 21–89 years). A total of 102 patients were male (64.5%), and 56 patients were female (35.4%). Of these, the median of the preoperative Karnofsky performance status (KPS) of 119 patients was 80 (range, 40–100), while the median of the KPS of 89 patients was at least 70 (89/119, 74.7%). The median survival time was 11.83 months (range, 0.16–88.07), and the overall survival status was “deceased” in 106 (67.0%) and “living” in 52 patients (32.9%) at the time of data deposition. Patients were monitored for tumor recurrence during the initial and maintenance therapies by using magnetic resonance imaging (MRI) or computed tomography (CT). Recurrent tumors were found in 13 patients. The validation data set, which consisted of 413 primary GBM patients whose data were deposited in the “Merged Cohort of LGG and GBM (TCGA, Cell 2016)” data set (Table 1, Supplementary Figure 1B), was used to confirm the results derived from the 158 patients in the GBM training data set. Multivariate analyses for OS according to age, gender, gender and age, vital status (at last follow-up), and KPS were performed in each data set (Table 1). Of these, the hazard ratio (HR) for age ≥50 was higher than that for age <50 (HR = 1.73, 95% confidence interval (CI) 2.23–2.76, and ^*^P = 0.0121 in the training data set; HR = 2.29, 95% CI 1.77–2.99, and ^*^P < 0.0001 in the validation data set), and the HR for the “living” status was lower than that for the “deceased” status (HR = 0.47, 95%CI 0.28–0.76, and ^*^P = 0.0014 in the training data set; HR = 7.64 × 10^–11^, 95%CI –0.03, and ^*^P < 0.0001 in the validation data set). The other results of multivariate analyses for OS were not consistent with each other because of the small number of samples and the biases.

**Table 1:** Patient characteristics of glioblastoma multiformes

Characteristics	Training data set (*N* = 158)					Validation data set (*N* = 413)					
	*N* (%)	Median (Min–Max)	Multivariate analysis for OS				*N* (%)	Median (Min–Max)	Multivariate analysis for OS		
			HR	(95% CI)	*P*–value				HR	(95% CI)	*P*–value
Age	158 (100)	Age: 60 (21–89)					413 (100)				
Age<50	33 (20.8)	OS days: 360 (13–1642)	1				107 (25.9)	OS days: 585 (45–2997)	1		
Age>50	125 (79.1)	OS days: 270 (5–1458)	1.73	(1.12–2.76)	0.0121^*^		306 (74.0)	OS days: 292.5 (3–3825)	2.29	(1.77–2.99)	<0.0001^*^
Gender	158 (100)						413 (100)	Age: 58 (10–88)			
Female	56 (35.4)	OS days: 273.5 (6–1458)	1				170 (41.0)	OS days: 288 (3–3825)	1		
Male	102 (64.5)	OS days: 314.5 (5–1642)	1.03	(0.73–1.54)	0.7699		243 (58.9)	OS days: 363 (3–3474)	1.20	(0.96–1.50)	0.1095
Gender and age											
Female	56 (100)	Age: 62 (21–85)				Female	170 (100)	Age: 57 (10–84)			
Age < 62	26 (46.4)	OS days: 356 (13–1458)	1			Age<57	83 (48.8)	OS days:417 (3–3825)	1		
Age > 62	30 (53.5)	OS days: 143.5 (6–1448)	1.83	(0.83–4.17)	0.1366	Age>57	87 (51.1)	OS days:240 (3–3615)	1.85	(1.3–2.64)	0.0006^*^
Male	102 (100)	Age: 60 (30–89)				Male	243 (100)	Age: 58 (14–88)			
Age < 60	49 (48.0)	OS days: 316 (5–1642)	1			Age<58	113 (46.7)	OS days:471 (6–2715)	1		
Age > 60	53 (51.9)	OS days: 298 (21–1228)	1.05	(0.63–1.74)	0.8578	Age>58	130 (53.2)	OS days:274.5 (3–3474)	1.94	(1.44–2.61)	<0.0001^*^
Vital status (last follow-up)	158 (100)	OS days: 285.5 (5–1642)					413 (100)	OS days: 339 (3–3825)			
Deceased	106 (67.0)	OS days: 331 (5–1458)	1				329 (79.6)	OS days: 372 (3–3825)	1		
Living	52 (32.9)	OS days: 269 (13–1642)	0.47	(0.28–0.76)	0.0014^*^		84 (20.3)	OS days: 243 (3–2778)	7.64E–11	( –0.03)	<0.0001^*^
KPS	119 (100)	KPS: 80 (40–100)					307 (100)	KPS: 80 (20–100)			
KPS < 70	30 (25.2)	OS days: 192 (26–1448)	1				70 (22.8)	OS days: 207 (6–1791)	1		
KPS > 70	89 (74.7)	OS days: 331 (13–1458)	0.93	(0.53–1.70)	0.7984		237 (77.1)	OS days: 432 (3–3825)	0.45	(0.33–0.62)	<0.0001^*^

Note: OS; overall survival, HR; hazard ratio, KPS; Karnofsky performance status. Asterisks (^*^); statistically significant.

### Identification of Th1 and Th2 scores associated with PD-L1/PD-1 activity

Expression data were obtained from 158 primary GBM patients from TCGA, which were derived post-RNA-Seq performed on the tissues obtained by tumor resections and biopsies. However, the tissues from which the samples were derived, e.g. from a central or peripheral tumor or from a hypoxic or normoxic region, are unknown. Nine genes involved in Th1 and Th2 cell functions and/or cancer immunotherapy were selected as predictors (Table 2 and Supplementary Figure 2). The scatter plot in Supplementary Figure 3A shows the associations between the estimated ensemble mortalities and expression levels of four selected genes (IL12RB1, IL12RB2, IFNG, and TBX21) for Th1 cells and two selected genes (IL4 and GATA3) for Th2 cells (Supplementary Figure 3B). The calculated Pearson’s correlation coefficients (*r*^2^) were not statistically significant (*P* > 0.05, log-rank test). Thus, the expression levels of these genes were independent and were thus used as a valid input for the calculation of Th1 and Th2 scores to estimate prognosis in GBM.

**Table 2:** Gene set for Th1/2 differentiation and immune checkpoint

				Statistics for expression (FPKM)				
Symbol	RefSeq	Description	Alias	Median	Average	Min	Max	95%CI for Ave
CD274	NM_014143	CD274 molecule	B7-H, B7H1, PD-L1, PDCD1L1, PDCD1LG1, PDL1	28.34	49.31	0	541.56	38.06–60.56
GATA3	NM_002051	GATA binding protein 3	HDR, HDRS	8.33	23.84	0	446.81	15.63–32.03
IFNG	NM_000619	Interferon gamma	IFG, IFI	0.00	1.83	0	181.95	-0.44–4.11
IL12RB1	NM_005535	Interleukin 12 receptor subunit beta 1	CD212, IL-12R-BETA1, IL12RB, IMD30	40.26	51.81	0	220.73	45.77–57.84
IL12RB2	NM_008354	Interleukin 12 receptor subunit beta 2	IL-12RB2, IL-12R-Beta-2	4.03	6.88	0	96.68	5.12–8.64
IL4	NM_000589	Interleukin 4	BCGF-1, BCGF1, BSF-1, BSF1, IL-4	0.00	0.30	0	4.35	0.19–0.40
PDCD1	NM_005018	Programmed cell death 1	CD279, PD-1, PD1, SLEB2, hPD-1, hPD-l, hSLE1	8.09	10.99	0	10.5.536	9.08–12.89
PDCD1LG2	NM_025239	Programmed cell death 1 ligand 2	B7DC, Btdc, CD273, PD-L2, PDCD1L2, PDL2, bA574F11.2	117.01	144.48	0	818.65	124.66–164.30
TBX21	NM_013351	T-box 21	T-PET, T-bet, TBET, TBLYM	5.34	9.30	0	77.87	7.43–11.17

Note: FPKM, fragments per kilobase of exon per million mapped sequence reads.

Study individuals were divided into high and low subgroups based on the median expression levels for IL12RB1, IL12RB2, IFNG, TBX21, IL4, and GATA3. The correlations between the expression of each gene and patient survival were not statistically significant (*P* > 0.05, log-rank test), except for GATA3 (^*^P = 0.0324) (Supplementary Figure 4). The Th1 and Th2 scores calculated as a combination of the expression values of these genes were used in estimating the prognosis of GBM. PCA was performed to compute the Th1 and Th2 scores as a linear combination of the four and two genes, respectively, based on the following formulas:

Th1 score = 0.7671 × TBX21 + 0.506 × IL12RB1 + 0.2899 × IFNG + 0.2673 × IL12RB2,

Th2 score = 1.0 × GATA3 + 0.0057 × IL4.

**Figure 1:**
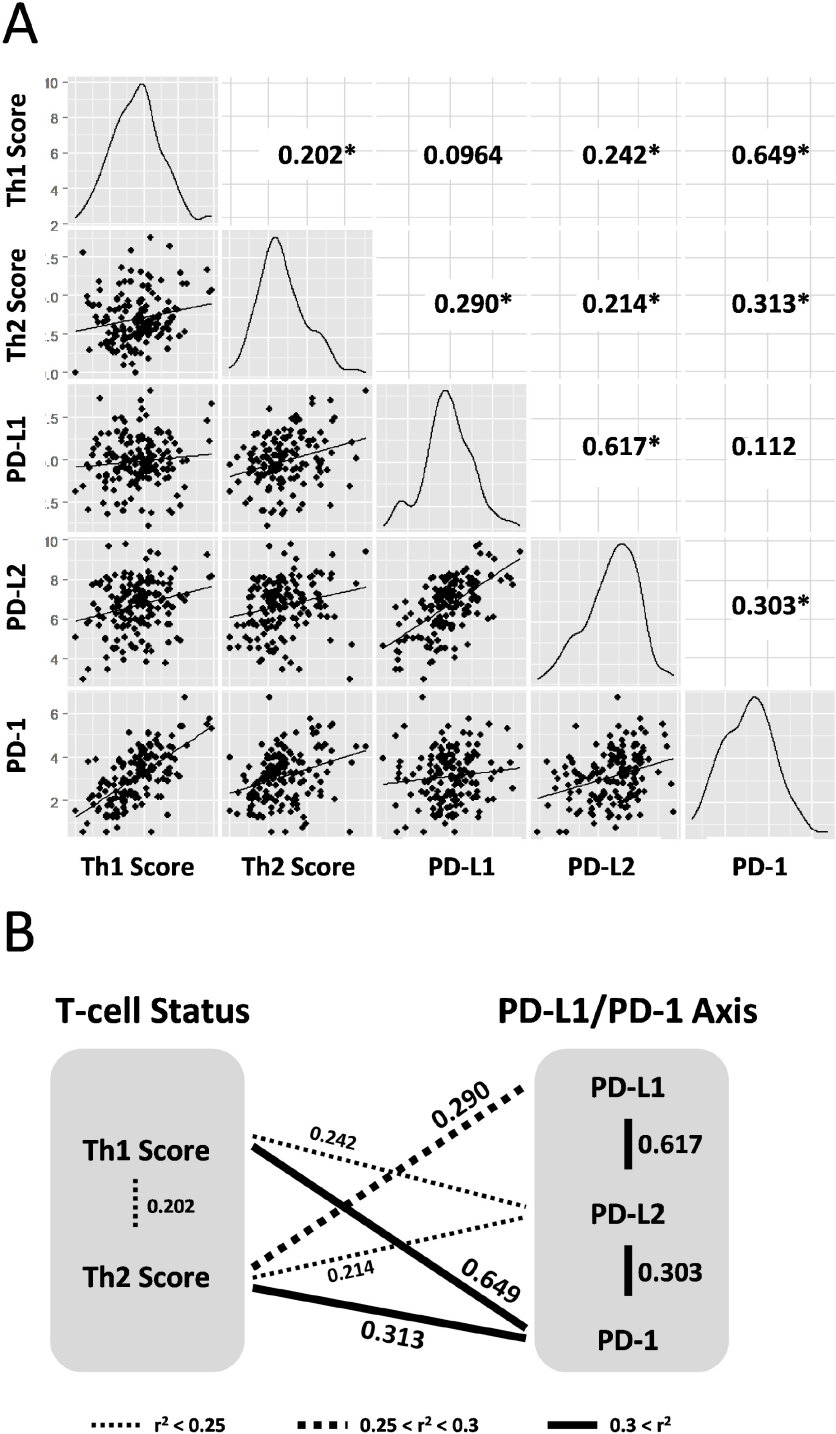
Th1 and Th2 helper T-cell scores are correlated to expression of PD-L1/PD-1 axis genes in 158 GBM training data set (**A**) Density plots and correlation coefficient values (*r*^2^) are presented in scatter plot matrices. Targeting molecules for immunotherapy; CD274/PD-L1, PDCD1LG2/PD-L2, and PDCD1/PD-1. Numbers indicate correlation coefficient values (*r*^2^). Asterisk indicates statistically significant (^*^P < 0.05, log-rank test). (**B**) Schematic representation of correlation between Th1 and Th2 helper T-cell status and PD-L1/PD-1 axis genes. Correlations with statistical significances are drawn with thin dotted lines, thick dotted lines, and thick lines as weak (*r*^2^ < 0.25), moderate (0.25 < *r*^2^ < 0.3), and strong (*r*^2^ > 0.3) correlations, respectively. Data were derived from the scatter plot matrices.

The Th1 and Th2 scores were derived from the expression values of the target genes (Supplementary Figures 1A and 2; derived from “Glioblastoma Multiforme (TCGA, Provisional)” data set) and were calculated using the above formula. The scatter plot shows the relationship between the Th1 and Th2 scores and expression values of the three genes (CD274 (PD-L1), PDCD1LG2 (PD-L2), and PDCD1 (PD-1)) that were considered as cancer immunotherapy genes involved in the PD-L1/PD-1 axis (Figure 1). All calculated r^2^ values were statistically significant to a certain degree (^*^P < 0.05, log-rank test) for Th1-PDCD1 (*r*^2^ = 0.649), CD274-PDCD1LG2 (*r*^2^ = 0.617), Th2-PDCD1 (*r*^2^ = 0.313), PDCD1-PDCD1LG2 (*r*^2^ = 0.303), Th2-CD274 (*r*^2^ = 0.290), Th1-PDCD1LG2 *r*^2^ = 0.242), Th2-PDCD1LG2 (*r*^2^ = 0.214), and Th1-Th2 (*r*^2^ = 0.202), except for Th1-CD274 and CD274-PDCD1 (*r*^2^ = 0.0964 and *r*^2^ = 0.112, respectively; *P* > 0.05) (Figure 1A). Summarizing the above results, the Th1 score was strongly and weakly correlated with the expression of PD-1 and PD-L2, respectively, but not with PD-L1 (Figure 1B). On the other hand, the Th2 score was moderately, weakly, and strongly correlated with PD-L1, PD-L2, and PD-1, respectively (Figure 1B). In addition, the PD-L1/PD-1 axis genes were strongly correlated with each other, whereas the Th1 and Th2 scores were weakly correlated (Figure 1B), suggesting that the Th1 and Th2 scores in GBM are correlated with the expression of genes in the PD-L1/PD-1 axis in a complex manner. Interestingly, a correlation between Th1 score and PD-L1 expression was not observed, implying that the difference between Th1 and Th2 scores is related to the PD-L1/PD-1 axis activity.

### Th1 and Th2 scores predicted the most significant survival curves in GBM

**Figure 2:**
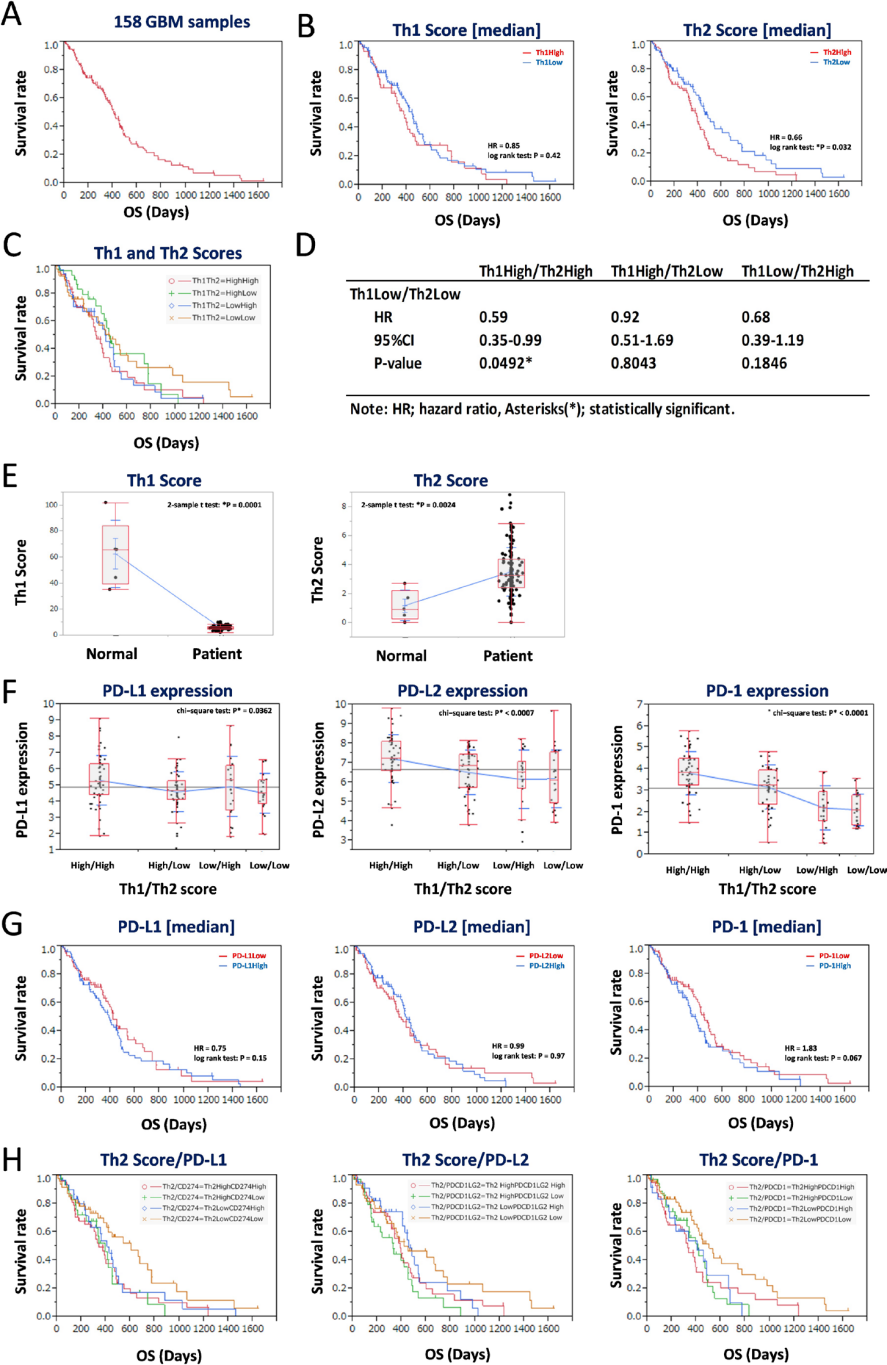
Low balance of Th2 score with lower expression of PD-L1/PD-1 axis genes estimates a good prognosis of 158 GBM patients in the training data set (**A**) Kaplan-Meier survival analysis of 158 GBM patients. (**B**–**C**) Kaplan-Meier survival analysis of GBM patients with Th1 score (left in B) and Th2 score (right in B), and Th1/Th2 score (C). The 158 GBM patients were divided by median of each score. (**D**) Statistics of overall survival of the Th1^Low^Th2^Low^ subgroup, compared with the other subgroups, shown in (C). HR: hazard ratio, 95% confidence interval (CI): lower-upper, log-rank test: *P*-value. Asterisks (^*^) indicates statistically significance. (**E**) Comparison of Th1 (left) and Th2 (right) scores in normal brain and GBM. (**F**) Differential expression of PD-L1 (left), PD-L2 (center), and PD-1 (right) in groups divided by median of Th1 and Th2 scores. (**G**) Kaplan-Meier survival analysis of GBM patients with expression of PD-L1 (left), PD-L2 (center), and PD-1 (right). The 158 GBM patients were divided by median of each expression. (**H**) Kaplan-Meier survival analysis of Th2^Low^ GBM patients with expression of PD-L1 (left), PD-L2 (center), and PD-1 (right). The 158 GBM patients were divided into four groups by each value of Th2 score and gene expression. HR indicates hazard ratio. ^*^P < 0.05 with log-rank test is statistically significant. OS, overall survival time (days).

The median OS of all 158 GBM patients was 285.5 days (range, 5–1642), and results of Kaplan-Meier analysis returned a median survival time of 678 days (Figure 2A). GBM patients were divided into two subgroups based on the median Th1 and Th2 scores, and Kaplan-Meier analysis was performed. The Th2^Low^ group, but not the Th1^Low^ group, showed better prognoses than the Th2^High^ group (^*^P = 0.032, log-rank test) (Figure 2B, left and right panels). Furthermore, we performed Kaplan-Meier and statistical analysis for each of the four combinations of the Th1 and Th2 subgroups (Th1^High^Th2^High^, Th1^High^Th2^Low^, Th1^Low^Th2^High^, and Th1^Low^Th2^Low^) (Figure 2C and 2D). Results showed that the Th1^Low^Th2^Low^ subgroup had the most significant good prognosis (Figure 2C), especially compared with the Th1^High^Th2^High^ subgroup (HR = 0.59, 95%CI 0.35–0.99, ^*^P = 0.0492) (Figure 2D). Compared with healthy brain specimens, the GBM specimens showed lower Th1 scores (Figure 2E, left panel). By contrast, the Th2 scores in the GBM specimens were higher than those in the healthy brain specimens (Figure 2E, right panel), indicating that a lower Th2 score is associated with good prognosis. Additionally, expression levels of CD274 (PD-L1), PDCD1LG2 (PD-L2), and PDCD1 (PD-1) were also significantly more downregulated in the Th2^Low^ subgroups, including the Th1^High^Th2^Low^ and Th1^Low^Th2^Low^ subgroups, than in the Th1^High^ subgroups. Notably, expression levels of the abovementioned genes were the lowest in the Th1^Low^Th2^Low^ subgroup (^*^P < 0.05, chi-square test; Figure 2F, left, center, and right panels). However, dividing the CD274^Low^, PDCD1LG2^Low^, and PDCD1^Low^ subgroups based on the median expression levels did not effectively divide the survival curves (*P* > 0.05, log-rank test; Figure 2G, left, center, and right panels). The Th2 score and expression levels of the PD-L1/PD-1 axis genes were then combined and again analyzed via Kaplan-Meier analysis (Figure 2H). The Th2^Low^ subgroup showed downregulated expression of CD274 (PD-L1), PDCD1LG2 (PD-L2), and PDCD1 (PD-1), which reliably divided the Kaplan-Meier curves and showed the best prognosis among all subgroups (Figure 2H, left, center, and right panels). These results suggested that a lower Th2 score and lower activity of the PD-L1/PD-1 axis could serve as good prognostic markers for GBM.

### Low Th2 score and low PD-L1/PD-1 activity predicted good GBM prognosis

**Figure 3:**
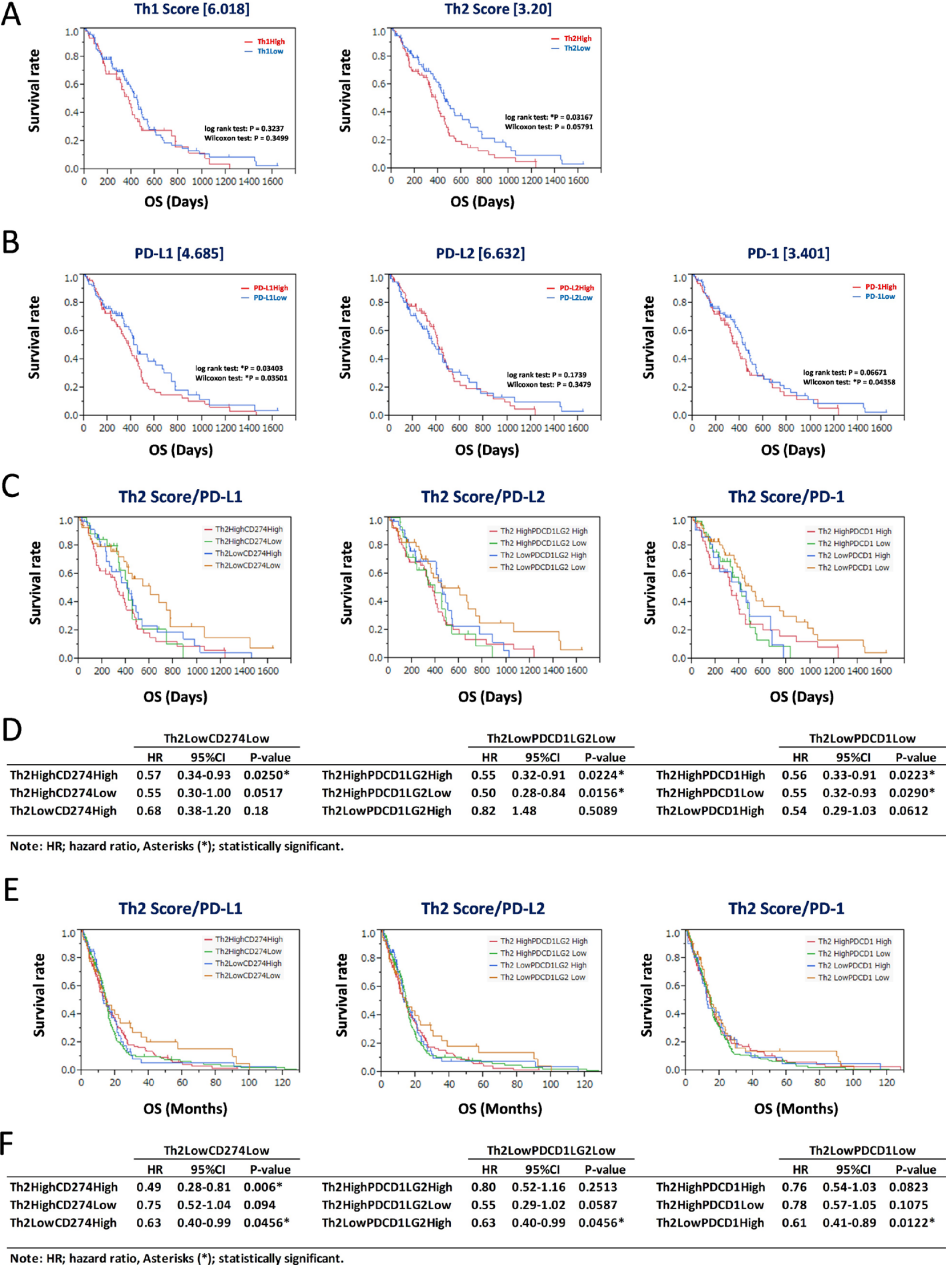
Assessment for prognostic markers in Th2^Low^ GBM patients with lower expression of PD-L1/PD-1 axis genes (**A**) Kaplan-Meier survival analysis of 158 GBM patients in the training data set with Th1 score (left) and Th2 score (right). The 158 GBM patients were divided by threshold. (**B**) Kaplan-Meier survival analysis of 158 GBM patients in the training data set with expression of PD-L1 (left), PD-L2 (center), and PD-1 (right). The 158 GBM patients were divided by threshold. (**C**–**F**) Kaplan-Meier survival analysis of Th2^Low^ GBM patients with expression of PD-L1 (left), PD-L2 (center), and PD-1 (right) in the 158 GBM training data set and the 413 GBM validation data set. The 158 GBM training data set (C) and the 413 GBM validation data set (E) were divided into four groups by each threshold of Th2 score and gene expression as follows: Th1 score = 6.018, Th2 score = 3.20, CD274/PD-L1 = 4.685, PDCD1LG2/PD-L2 = 6.632, and PDCD1/PD-1 = 3.401. ^*^P < 0.05 with log-rank test is statistically significant. OS, overall survival time (days). Statistics of results of overall survival for Th2/PD-L1 axis genes with thresholds in the 158 GBM training data set (D) and the 413 GBM validation data set (F). HR: hazard ratio, 95% confidence interval (CI): lower-upper, log-rank test: *P*-value. Asterisks (^*^) indicates statistically significance.

Th1 and Th2 scores and expression levels of CD274 (PD-L1), PDCD1LG2 (PD-L2), and PDCD1 (PD-1) were assessed for prediction of GBM prognosis. The Th1 and Th2 subgroups were divided based on thresholds of 6.018 and 3.20, respectively (Figure 3A). The Th2^Low^ subgroup predicted good prognosis based on Kaplan-Meier analysis (^*^P = 0.03167, log-rank test; Figure 3A, right panel), whereas the result for the Th1^Low^ subgroup was not significant (*P* = 0.3237, log-rank test; Figure 3A, left panel). Similarly, GBM patients were divided according to the FPKM values of CD274 (4.685), PDCD1LG2 (6.632), and PDCD1 (3.401) (Figure 3B). CD274^Low^ and PDCD1^Low^ subgroups were associated with good prognosis (^*^P = 0.03501 and ^*^P = 0.04358, respectively, Wilcoxon test; Figure 3B, left and right panels), whereas the result for PDCD1LG2^Low^ was not significant (*P* = 0.3479, Wilcoxon test; Figure 3B, center panel). Furthermore, the Th2^Low^ subgroup, which showed downregulation of CD274 (PD-L1), PDCD1LG2 (PD-L2), and PDCD1 (PD-1), was associated with the best prognosis among all subgroups (Figure 3C, left, center, and right panels). Statistical analyses showed that the hazard ratios of Th2^Low^CD274^Low^, Th2^Low^PDCD1LG2^Low^, and Th2^Low^PDCD1^Low^ tended to be lower than those of the other subgroups, with statistical significance (HR < 0.57, ^*^P < 0.0290) in each analysis (Figure 3D). This tendency was also confirmed in the 413 patients of the GBM validation data set (Table 1, Figure 3E, and Supplementary Figure 1B; derived from the “Merged Cohort of LGG and GBM (TCGA, Cell 2016)” data set). However, in each analysis in the validation data set, the difference between the training and validation data sets and the bias in data slightly modified the statistical significance of the Th2^Low^ subgroups with lower expression of PD-L1/PD-1 axis genes compared with the other subgroups (HR < 0.63, ^*^P < 0.0456; Figure 3F). Nevertheless, it was confirmed that the Th2^Low^ subgroup with lower expression of PD-L1, PD-L2, and PD-1 was associated with the best prognosis among all the subgroups in the two independent GBM data sets. On the other hand, Th1 scores based on PD-L1, PD-L2, and PD-1 expression did not effectively divide the survival curves in GBM (Supplementary Figure 5). These results suggest that the Th2 score and expression levels of CD274 (PD-L1) and PDCD1 (PD-1) are reliable estimators of GBM prognosis.

### Candidate pathways associated with patient survival in the PD-L1/PD-1 axis based on Th2 balance

**Figure 4:**
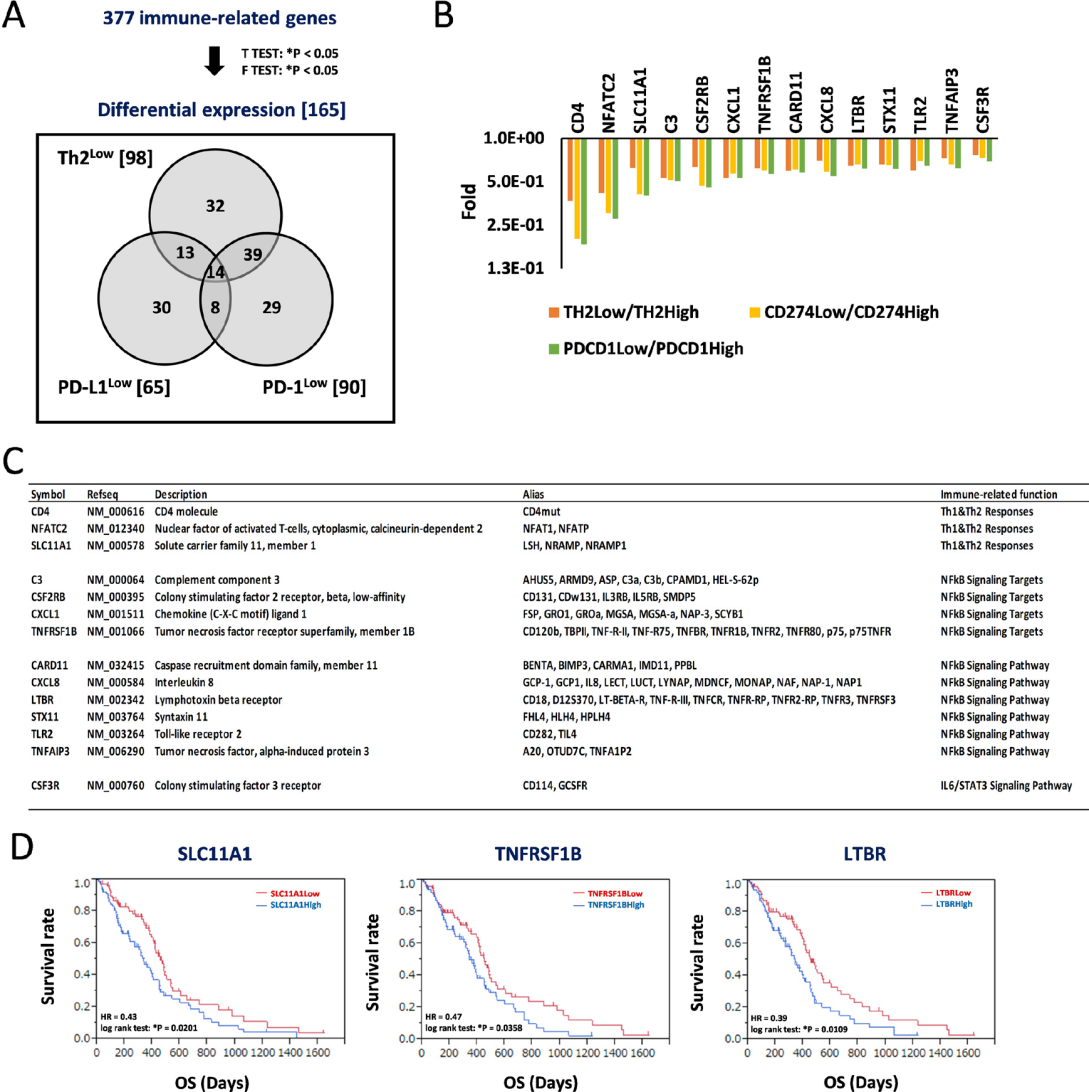
Decrease of signature genes for helper T-cell response and predominant inflammation signaling in Th2^Low^/PD-L1^Low^/PD-1^Low^ estimates a good prognosis in patients with GBM (**A**) Differential expression of immune-related genes estimates a good prognosis in patients with GBM. 377 immune-related genes were tested by *t*-test (^*^P < 0.05) and *F*-test (^*^P < 0.05), and 165 differential expression genes were identified, those expression were classified within Th2^Low^, PD-L1^Low^, and PD-1^Low^ subgroups, compared with each High subgroup, respectively. Numbers in parentheses and Venn diagram are the gene numbers in the set and subset, respectively. (**B**) Differential expression of 14 genes in the Th2^Low^/PD-L1^Low^/PD-1^Low^ subgroup. Fold differences of the gene expression in Low subgroups, compared with High subgroups, respectively, are shown in graph. (**C**) Characterization of 14 signature genes in Th2^Low^/PD-L1^Low^/PD-1^Low^. (**D**) Kaplan–Meier analysis for correlation between patient survivals and expression of SLC11A1 (left), TNFRSF1B (center), and LTBR (right). The 158 GBM samples were divided by median of each expression. HR indicates hazard ratio. ^*^P < 0.05 with log-rank test is significant. OS, overall survival time (days).

We next aimed to identify additional candidate biomarkers to achieve a more accurate estimation of prognosis in GBM patients. We repeated the analysis using RNA-Seq data deposited in TCGA. We extracted gene expression data of 158 GBM patients, identified 377 immune-related genes, and identified the differentially expressed genes. The analysis returned 165 differentially expressed genes after comparing gene expressions in the Th2^Low^ subgroup with those in the Th2High subgroup (98 genes), CD274^Low^ subgroup with those in the CD274^High^ subgroup (65 genes), and PDCD1^Low^ subgroup with those in the PDCD1^High^ subgroup (90 genes); the genes are presented in a Venn diagram (Figure 4A). Of these, 14 genes were classified into the Th2^Low^, CD274^Low^, and PDCD1^Low^ subgroups. Interestingly, all the 14 genes were downregulated in the Th2^Low^, CD274^Low^, and PDCD1^Low^ subgroups compared with those in the corresponding high subgroups (Figure 4A and 4B). Immune-related functions of the 14 genes included Th1 and Th2 responses (3 genes), nuclear factor-kappa B (NF-κB) signaling pathway (6 genes) and their corresponding targets (4 genes), and IL-6/signal transduction and activator of transcription 3 (STAT3) signaling pathway (1 gene), providing strong evidence that NF-κB signaling-related genes (Figure 4C). Furthermore, Kaplan-Meier analysis was used to divide the data into solute carrier family 11 member 1 (SLC11A1)^Low^ (HR = 0.43, ^*^P = 0.0201, log-rank test), tumor necrosis factor receptor superfamily member 1B (TNFRSF1B)^Low^ (HR = 0.47, ^*^P = 0.0358), and lymphotoxin beta receptor (LTBR)^Low^ (HR = 0.39, ^*^P = 0.0109) subgroups based on the median expression levels. These were found to predict good prognosis compared to each corresponding high subgroup (Figure 4D and Supplementary Figure 7). Thus, future studies should investigate the correlations among Th2 scores, the PD-L1/PD-1 axis, and the expression profiles based on the 14 candidate genes, especially SLC11A1, TNFRSF1B, and LTBR, as well as how these parameters can be used to effectively predict prognoses of GBM patients.

In addition, we also examined the changes in expression of 18 genes related to tumor-infiltrating macrophages (CD163, ITGAM, MRC1, and NCAM1), myeloid cells (ARG1, CCL2, CCR2, CD68, CSF1R, CXCL8, CXCR2, and IDO1), and natural killer (NK) cells (FCGR3A, FCGR3B, KLRA1P, KLRC1, KLRD1, and NCR1) in subgroups divided by the Th1 score, the Th2 score, and a combination of the two, compared with the median expression of each gene (Supplementary Figure 6A). The expressions indicated changes of only 0.87-fold to 1.30-fold and were not significant (*P* > 0.05, one-way ANOVA; Supplementary Figure 6B), suggesting that the tumor environments, including macrophages, myeloid cells, and NK cells, might have very little effect on the experiments.

**Figure 5:**
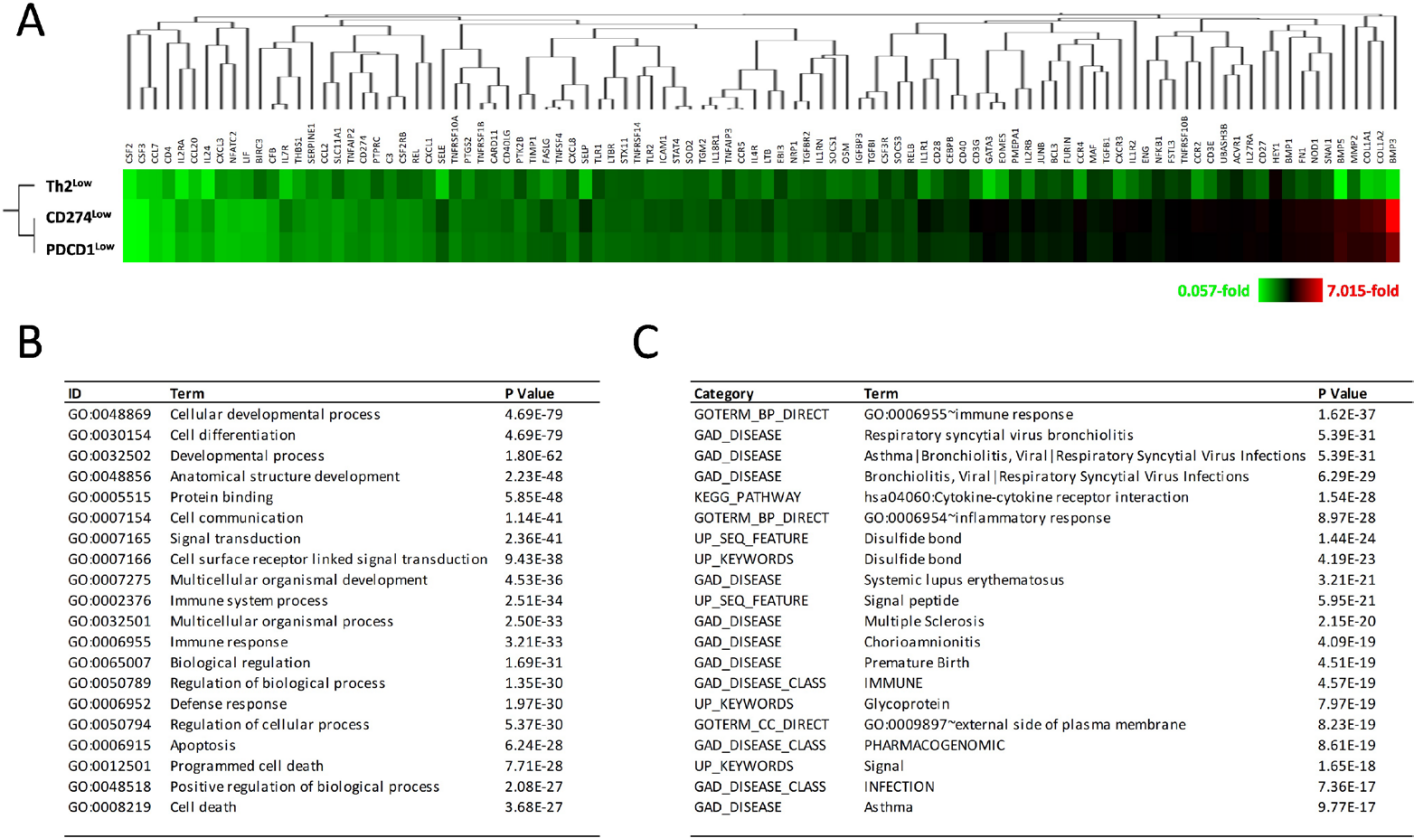
Decrease of gene expression in development, cell differentiation, immune response, and cell death in Th2^Low^ GBM patients (**A**) Decreased expression of 98 immune-related genes in Th2^Low^ GBM patients, associated with expression in CD274^Low^ and PDCD1^Low^ GBM patients. Fold differences are shown in heatmap with clustering analysis. Color configuration indicates 0.057-fold (green) to 7.015-fold (red), compared with each High subgroup. (**B**) Gene ontology analysis of 98 immune-related genes decreased in the Th2^Low^ subgroup. Representative GO terms with GOstat are presented. (**C**) Functional annotation of the immune-related genes decreased in Th2^Low^. Representative Terms with DAVIDv6.8 are presented.

On the other hand, all 98 differentially expressed genes in the Th2^Low^ subgroup showed the same magnitudes of downregulation as those in the Th2^High^ subgroup, but not in both the CD274^Low^ and PDCD1^Low^ subgroups, when compared to their corresponding high subgroups (Figure 5A), suggesting that the Th2 score is prior to CD274 and PDCD1 expression for prognostic prediction in GBM. Gene ontology (GO) analysis and functional annotation assigned 98 genes into GO terms, including immune response, cell differentiation, cellular developmental process, apoptosis, and cell death (^*^P < 3.68 × 10^–27^; Figure 5B). For the disease category, the differentially expressed genes were classified under the terms arthritis, asthma, atherosclerosis, bronchiolitis, chorioamnionitis, multiple myeloma, multiple sclerosis, systemic lupus erythematosus, and type 2 diabetes (^*^P < 9.77 × 10^–17^; Figure 5C). These results suggest that, in addition to immune responses, the 14 differentially expressed genes detected in Th2^Low^ GBM specimens were also involved in cell death and apoptosis, cancers, immune diseases, and infections. Thus, the suppression of NF-κB signaling, which is associated with complex diseases, can improve prognoses of GBM patients. A combined evaluation incorporating the Th1 and Th2 scores, expression levels of genes involved in the PD-L1/PD-1 axis, and the 14 novel candidate genes involved in Th1 and Th2 responses, NF-κB signaling, and IL6/STAT3 signaling could be used to derive more accurate estimates of the prognoses of GBM patients.

## DISCUSSION

In this study, we calculated scores that measure the balance between Th1 and Th2 cells based on the expression of PD-L1, PD-L2, and PD-1 and clinical information of 158 GBM patients deposited in TCGA and analyzed their prognoses. The results revealed that GBM patients with good prognoses have low Th2 balance based on IL4 and GATA3 expression and low expression of PD-L1 and PD-1. Downregulated expression of the PD-L1/PD-1 axis genes in Th2 cells was associated with good prognosis. Furthermore, 14 genes were identified as potential prognostic markers associated with Th1 and Th2 responses and major inflammatory signaling pathways, including NF-κB and IL-6/STAT3 signaling: SLC11A1, TNFRSF1B, and LTBR were found to be especially promising predictors of the prognoses of GBM patients and could be used to develop effective GBM treatment strategies. SLC11A1, TNFRSF1B, and LTBR have been known in the immune signature or immune environment, such as Th1/Th2 response and/or NF-κB signaling in tumors. However, detailed experiments and precise explanation on the molecular network and integrated function among these three genes, Th1/Th2 balance, and PD-L1/PD-1 axis should be done in the future study. SLC11A1, TNFRSF1B, and LTBR have not yet been reported on prognostic factors in GBM, thus, suggestive of novel target candidates for cancer immunotherapy. In general, a prognostic prediction could be useful for a treatment regimen, whereas how this can be applied in the clinical management would be a next subject beyond the study.

### Immune checkpoint regulation by cell signaling and transcription factors

IFN-γ, which is produced by Th1 T-cells, promotes the activities of type I interferons IFN α and IFN-β against tumor growth. IFN signaling is constitutively active in gliomas as well. However, the silencing of IFN-α/β receptors 1 or 2 (IFNAR1 or IFNAR2), which constitute a loop to IFN-α signaling, causes a reduction in PD-L1 and MHC class I/II expression in glioma cells, as well as an enhanced susceptibility to immune cell lysis of NK cells. Thus, the above findings suggested that autocrine IFN-α signaling, but not IFN-β or IFN-γ signaling, contributes to the immune evasion of gliomas [18]. Similarly, blockade of both the PD-L1 and CTLA-4 immune checkpoints substantially improved the efficiency of stimulatory cancer immunotherapies against GBM, which are mediated by the binding of thymidine kinase (TK)/Fms-like tyrosine kinase ligand (Flt3L), and in turn inhibit the activity of immunosuppressive myeloid cells in glioma microenvironments [19]. However, our results demonstrated that a lower proportion of Th2 cells, but not Th1 cells, which was associated with the downregulation of PD-L1/PD-1 axis genes, predicted good prognoses in GBM patients. Thus, combining the estimates of Th cell status and expression of PD-L1/PD-1 axis genes is important for making reliable prognostic predictions in GBM. We also identified SLC11A1 as a novel candidate prognostic marker for Th1 and Th2 responses, as demonstrated by the longer survival of the SLC11A1^Low^ subgroup compared to that of the SLC11A1^High^ subgroup, thereby indicating the role of Th cell status in GBM.

In addition, sustained expression of PD-1 has been observed in exhausted CD8^+^ T-cells in cancer and chronic viral infection. PD-1 expression is partly regulated by an exhaustion-specific enhancer that contains the retinoic acid receptor (RAR), TBX21 (T-bet), and SRY-related HMG-box 3 (SOX3) [20]. Similarly, targeted therapies for immune checkpoint blockade were found to be effective against tumors, whereas glial tumors in children require the SOX2 transcription factor, an embryonic neural stem cell antigen that is strongly implicated in the biology of glioma-initiating cells and acts as an antigenic molecule in anticancer immunity [21, 22]. Although other transcription factors, including TP53 tumor suppressor and KRAS proto-oncogene in lung adenocarcinoma, are involved in PD-1 blockade immunotherapy [23], the correlation between lower NF-κB levels and GBM prognosis represents a potential therapeutic pathway by targeting the PD-L1/PD-1 axis and has also been reported in breast cancer [24]. In addition to the known transcription factor pathways, combined treatment via immune checkpoint blockade against PD-L1/PD-1 axis and suppression of NF-κB signaling targeting TNFRSF1B and/or LTBR could serve as an effective therapeutic strategy.

### Influence of other T-cell subsets, including Th17, Treg, and CD8^+^ cells, on GBM prognosis

Th17 cells are a subset of pro-inflammatory helper T-cells defined by the production of IL-17 [25]. Th17 cells are derived from CD4+ cells and are involved in regulatory T-cells (Tregs) [26, 27]. TBX21 (T-bet), GATA3, and retinoic acid receptor (RAR)-related orphan receptor gamma thymus (RORγt; encoded by the RORC gene) stimulates CD4^+^ cells, which is differentiated into Th17 cells [26]. Th17 cells produce IL-2 that is required for generation and maintenance of Tregs but inhibits the Th17 cell differentiation [27]. However, Th1 and Th2 lineages are developmentally different from the Th17 lineage [28]. In this study, we only detected colony stimulating factor 3 receptor (CSF3R), which is related to the IL6/STAT3 signaling pathway, as a differentially expressed gene in the Th2^Low^, PD-L1^Low^, and PD-1^Low^ subgroups compared with the corresponding high subgroups (Figure 4A–4C); the subgroup divided by CSF3R expression did not show statistical significance for prognosis in the 158 GBM patients (HR = 0.96, *P* = 0.34, log-rank test; Supplementary Figure 7K). Furthermore, the signature factors of Th17 cells, such as IL-6, IL-21, IL-23, and ROR-γ, were not detected among the 98 differentially expressed genes in the Th2^Low^ subgroup. Therefore, whether the Th1/Th2 balance is correlated with Th17 cells in GBM should be more addressed in further studies.

On the other hand, TGF-β pathway genes, related to Treg differentiation [29,30], including TGFB1, TGFBR2, bone morphogenetic protein (BMP) family genes, including BMP1, BMP3, and BMP5, and activin A receptor type I (ACVR1) were detected as differentially expressed genes in the Th2^Low^ subgroup. Interestingly, all of these genes were downregulated in the Th2^Low^ subgroup compared with the Th2^High^ subgroup (Figure 5A), suggestive of a positive correlation between the Th2^Low^ subgroup and Treg differentiation. In the context of these results, the Th2 and Th17 cell subsets may be weakly correlated in GBM. Because lower expression of GATA3 contributing the low score of Th2 also deregulate Th17 differentiation from CD4^+^ cells [26], and further, dysregulation of Th17 cells also cause malfunctions of Treg via lower levels of signaling pathways of TGF-β superfamily in Th2^Low^ gene profiling [29,30]. In addition, CD4 was downregulated in the Th2^Low^ subgroup (Figure 5A), whereas CD8 was not included in the 98 differentially expressed genes in the Th2^Low^ subgroup, suggesting that CD8 expression and/or CD8^+^ cells may not have been correlated with the low Th2 balance in the GBM patients and their long overall survivals.

## MATERIALS AND METHODS

### Data set

Clinical information and RNA-Seq gene expression data were obtained from The Cancer Genome Atlas (TCGA) (NIH, https://cancergenome.nih.gov/) deposited between 2008 and 2012. Out of 604 GBM clinical samples, 166 samples with available clinical information and RNA-Seq data were downloaded. Duplicate samples, such as those from recurrent tumors, were removed from the analysis. The final data set comprised 158 samples available for survival distribution analysis and gene expression profiling, as a training data set. And also, 1120 low grade glioma (LGG) and GBM data were downloaded. 558 LGG samples and duplicated samples, such as identical 145 GBM samples including into the training data set as described above, were removed. Finally, out of 417 GBM samples, 413 samples with available clinical information and RNA-Seq data were remained, as a validation data set.

### Principal component analysis (PCA)

PCA was used to classify GBM patients into subgroups and to estimate patient prognosis in a simple form, such as a linear combination of expression values of target genes and estimates of T-cell status, as previously described [31,32]. PCA was performed using normalized values of fragments per kilobase of exon per million mapped sequence reads (FPKM) of target genes using a multivariate analysis tool for PCA using built-in modules in JMP (SAS Institute Inc., Tokyo, Japan).

### Kaplan-Meier analysis

The Kaplan-Meier method was used to estimate survival distributions for each group with log-rank test among subgroups using JMP built-in modules (SAS Institute Inc., Tokyo, Japan). Hazard ratios (HR) and 95% confidence intervals (CI) were calculated based on a logistic regression model with respect to clinical variables that were assessed via multivariate analysis with stepwise selection to compare groups. Overall survival (OS) was defined as the date of diagnosis of GBM to the date of death or last follow-up.

### Clustering analysis and gene ontology (GO) analysis

Clustering was performed using a hierarchical clustering method using JMP built-in modules (SAS Institute Inc., Tokyo, Japan). Functional GO annotation was performed using GOstat [33] and The Database for Annotation, Visualization and Integrated Discovery (DAVID) v6.8 [34].

### Statistics

Statistical analyses were performed using R software [35], Bioconductor [36], and JMP v10 (SAS Institute Inc., Tokyo, Japan). Statistical significance was assessed using a log-rank test, two-sample *t*-test, chi-square (χ^2^) test, one-way analysis of variance (ANOVA), and Wilcoxon/Kruskal-Wallis test as appropriate. P^*^ < 0.05 was considered statistically significant.

## SUPPLEMENTARY MATERIALS FIGURES


