# Supplementary material for: Correlation between lower balance of Th2 helper T-cells and expression of PD-L1/PD-1 axis genes enables prognostic prediction in patients with glioblastoma
Source: Oncotarget. 2018 Apr 10;9(27):19065–78. doi: 10.18632/oncotarget.24897 (PMC5922378; doi:10.18632/oncotarget.24897)
Supplement: Supplementary file 1 [file oncotarget-09-19065-s001.pdf]

Correlation between lower balance of Th2 helper T-cells and expression of PD-L1/PD-1 axis genes enables prognostic prediction in patients with glioblastoma

SUPPLEMENTARY MATERIALS

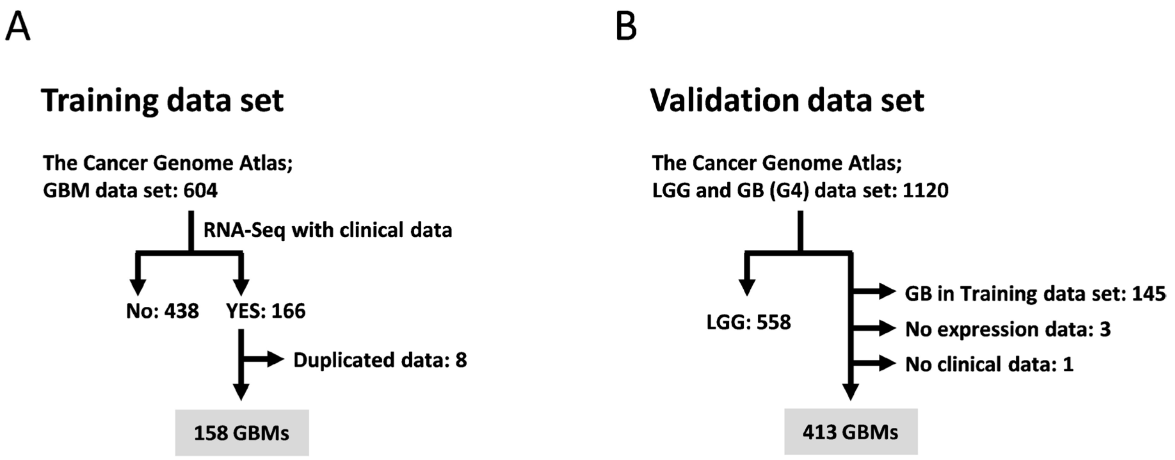

Supplementary Figure 1: Construction of the training data set and validation data set for GBMs derived from TCGA database in the study. (**A**) The training data set for the GBM study. (**B**) The validation data set for the GBM study. Numbers indicate sample numbers. The training data set were first examined, and furthermore, the validation data set were also used for confirmations for results derived from the training data set.

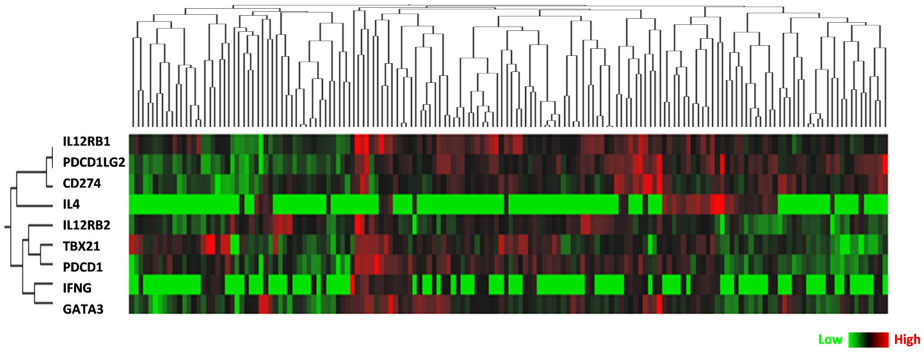

Supplementary Figure 2: Expression profile of genes related to Th1 and Th2 helper T-cells and the PD-L1/PD-1 immune checkpoint in 158 patients with GBM. Relative gene expression was shown in heat map with clustering analysis. Color configuration indicates low (green) to high (red).

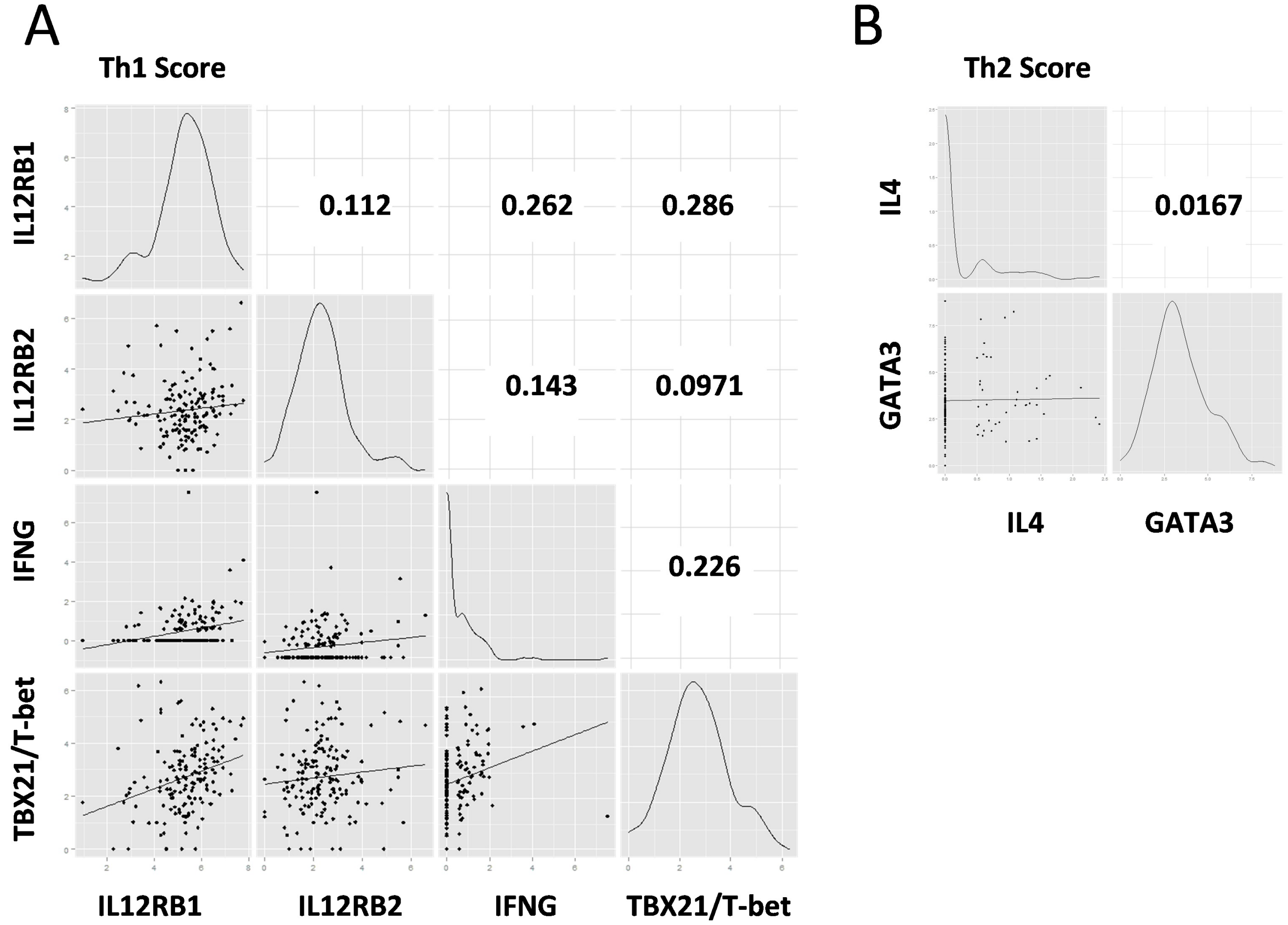

Supplementary Figure 3: Correlation among gene expression of regulatory molecules for Th1 and Th2 helper T-cells in GBM. (**A**) Th1 score: IL12RB1, IL12RB2, IFNG, and TBX21/T-bet. (**B**) Th2 score: IL4 and GATA3. Density plots and correlation coefficient values are presented in scatter plot matrices. Numbers indicate correlation coefficient. Not statistically significant (*P* > 0.05, log-rank test).

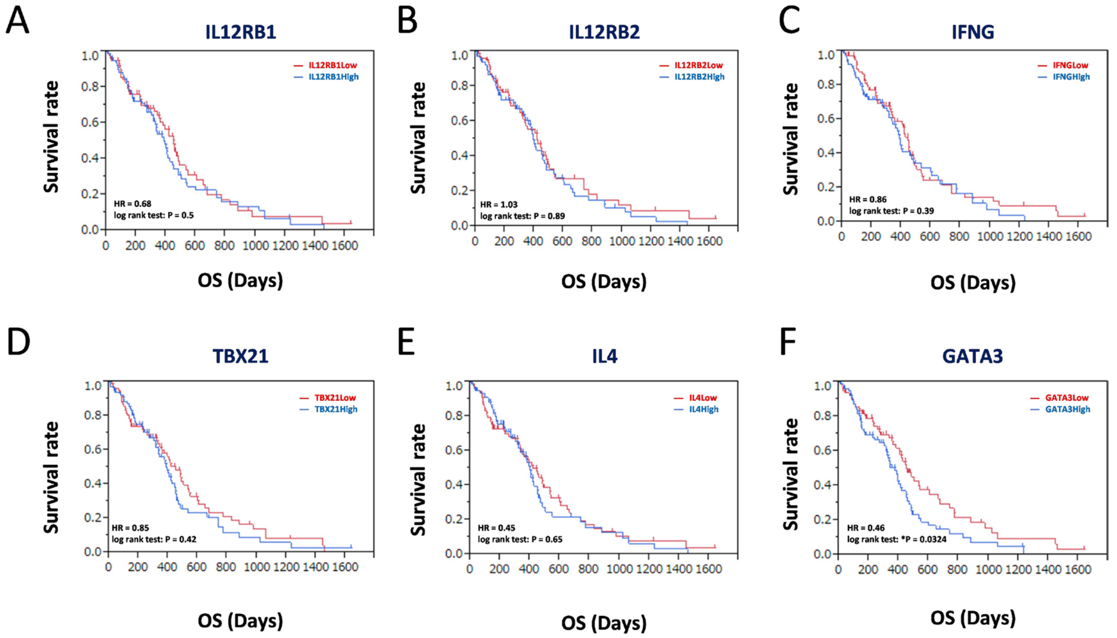

Supplementary Figure 4: Kaplan-Meier survival analysis of genes used by evaluating Th1 and Th2 helper T-cell status in patients with GBM. The 158 GBM samples were divided with each expression of (**A**) IL12RB1, (**B**) IL12RB2, (**C**) IFNG, (**D**) TBX21, (**E**) IL4, and (**F**) GATA3. HR indicates hazard ratio. $^{*}P < 0.05$ with log-rank test is statistically significant. OS, overall survival time (days).

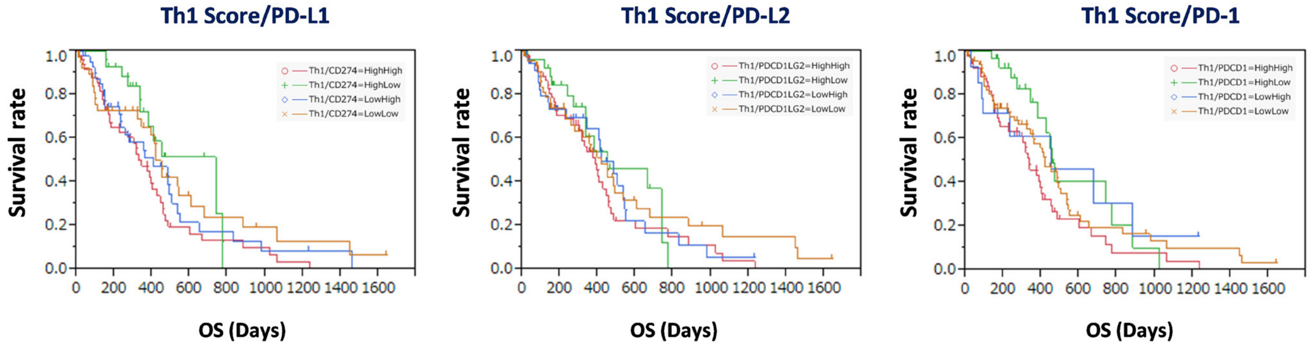

Supplementary Figure 5: Kaplan-Meier survival analysis for the expression of PD-L1 (left), PD-L2 (center), and PD-1 (right) on the Th1 score in GBM. The 158 GBM patients were divided into four groups by each threshold as follows: Th1 score = 6.018, CD274/PD-L1 = 4.685, PDCD1LG2/PD-L2 = 6.632, and PDCD1/PD-1 = 3.401. OS, overall survival time (days).

A

Symbol	Refseq	Description	Alias	Cell type
CD163	NM_004244	CD163 molecule	M130, MM130, SCARI1	Tumor-infiltrating macrophage
ITGAM	NM_000632	Integrin subunit alpha M	CD11B, CR3A, MAC-1, MAC1A, MO1A, SLEB6	Tumor-infiltrating macrophage
MRC1	NM_002438	Mannose receptor C-type 1	CD206, CLEC13D, CLEC13DL, MMRL1	Tumor-infiltrating macrophage
NCAM1	NM_000615	Neural cell adhesion molecule 1	CD56, MSK39, NCAM	Tumor-infiltrating macrophage
ARG1	NM_000045	Arginase 1	arginase 1	Tumor-infiltrating myeloid
CCL2	NM_002982	C-C motif chemokine ligand 2	GDCF-2, HC11, HSMCR30, MCAF, MCP-1, MCP1, SCYA2, SMC-CF	Tumor-infiltrating myeloid
CCR2	NM_001123041	C-C motif chemokine receptor 2	CC-CKR-2, CCR-2A, CCR2B, CD192, CKR2, CKR2A, CKR2B, CMKBR2, MCP-1-R	Tumor-infiltrating myeloid
CD68	NM_001251	CD68 molecule	GP110, LAMP4, SCARD1	Tumor-infiltrating myeloid
CSF1R	NM_001288705	Colony stimulating factor 1 receptor	C-FMS, CD115, CSF-1R, CSFR, FIM2, FMS, HDLS, M-CSF-R	Tumor-infiltrating myeloid
CXCL8	NM_000584	C-X-C motif chemokine ligand 8	GCP-1, GCP1, IL8, LECT, LUCT, LYNAP, MDNCF, MONAP, NAF, NAP-1, NAP1	Tumor-infiltrating myeloid
CXCR2	NM_001168298	C-X-C motif chemokine receptor 2	CD182, CDw128b, CMKAR2, IL8R2, IL8RA, IL8RB	Tumor-infiltrating myeloid
IDO1	NM_002164	Indoleamine 2,3-dioxygenase 1	IDO, IDO-1, INDO	Tumor-infiltrating myeloid
FCGR3A	NM_000569	Fc fragment of IgG receptor IIIa	CD16, CD16A, FCG3, FCGR3, FCGRIII, FCR-10, FCRIII, FCRIIIA, IGFR3, IMD20	Natural killer cells
FCGR3B	NM_000570	Fc fragment of IgG receptor IIIb	CD16, CD16b, FCG3, FCGR3, FCR-10, FCRIII, FCRIIIb	Natural killer cells
KLRA1P	NR_028045	Killer cell lectin like receptor A1, pseudogene	KLRA1, KLRAP1, LY49L, Ly-49L, Ly49	Natural killer cells
KLRC1	NM_001304448	Killer cell lectin like receptor C1	CD159A, NKG2, NKG2A	Natural killer cells
KLRD1	NM_001114396	Killer cell lectin like receptor D1	CD94	Natural killer cells
NCR1	NM_001145457	Natural cytotoxicity triggering receptor 1	CD335, LY94, NK-p46, NKP46	Natural killer cells

B

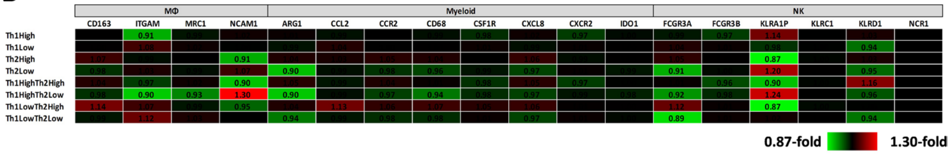

Supplementary Figure 6: Expression patterns of molecular markers for natural killer cells, macrophages, and myeloid. (**A**) List of molecular markers for macrophages (MΦ), myeloid, and natural killer (NK) cells. (**B**) Fold differences of genes in subgroups associated with Th1 Score, Th2 Score, and combination of Th1 and Th2 Scores, compared with the average of those in total 158 GBMs, are shown in the heat map. Color configurations indicate 0.87-fold (green) to 1.30-fold (red), compared with the average. Min; 0.87-fold, Max; 1.30-fold, $P > 0.05$; not significant (n.s.).

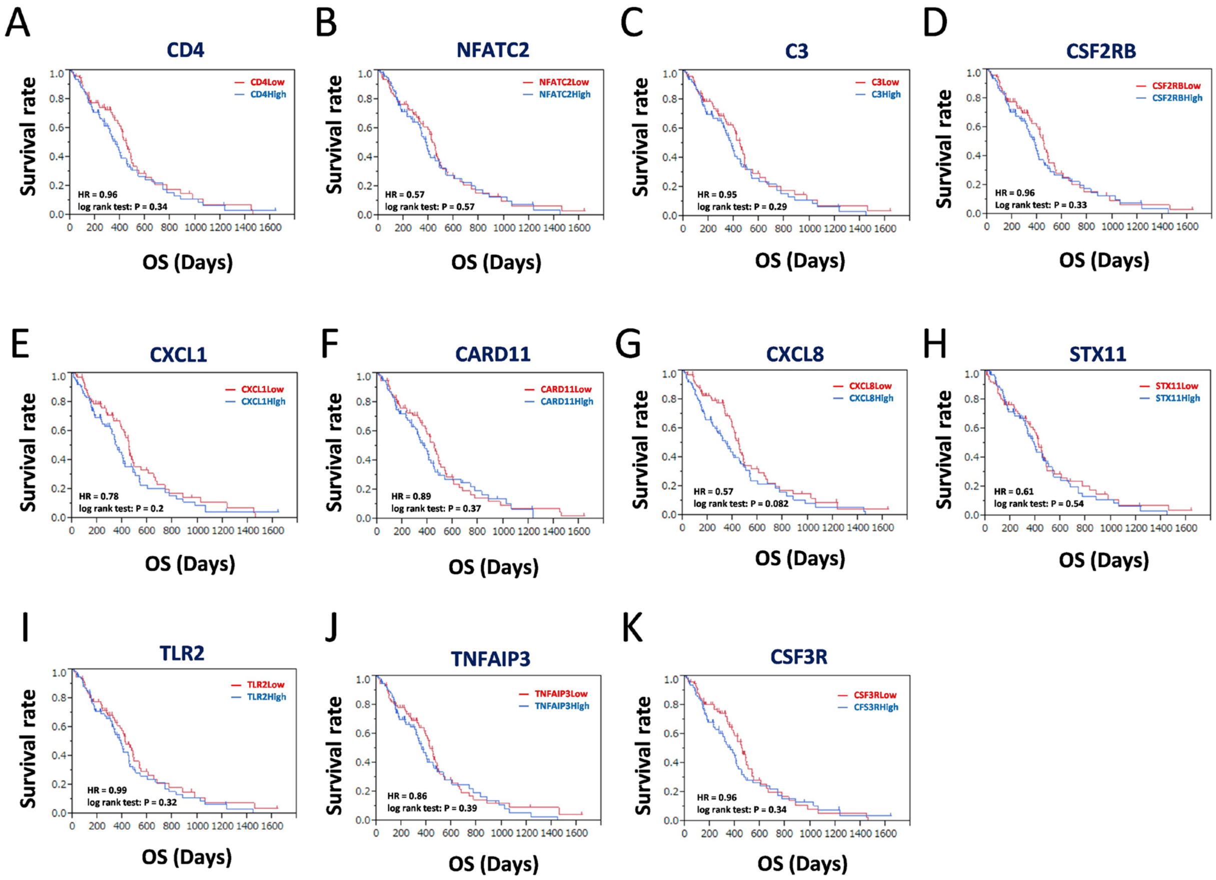

Supplementary Figure 7: Correlation between the expression of genes related to Th1 and Th2 response, NF-κB signaling, and IL6/STAT3 signaling, and prognosis in GBM. The 158 GBM samples were divided into two subgroups by median of each expression for (**A**) CD4 and (**B**) NFATC2 for Th1/Th2 response-related genes, (**C**) C3, (**D**) CSF2RB, (**E**) CXCL1, (**F**) CARD11, (**G**) CXCL8, (**H**) STX11, (**I**) TLR2, and (**J**) TNFAIP3 for NF-κB signaling-related genes, and (**K**) CSF3R for IL6/STAT3 signaling-related gene. HR indicates hazard ratio. Not statistically significant ($P > 0.05$, log-rank test). OS, overall survival time (days).
